# Clustering of Polymorphic Membrane Protein E Clade in *Chlamydia trachomatis* Lineages from Men Who Have Sex with Men

**DOI:** 10.3201/eid3010.240852

**Published:** 2024-10

**Authors:** Morika Mitobe, Hiroaki Kubota, Kai Kobayashi, Hirofumi Miyake, Misao Takano, Daisuke Mizushima, Hiroyuki Gatanaga, Shinichi Oka, Jun Suzuki, Kenji Sadamasu

**Affiliations:** Author affiliations: Tokyo Metropolitan Institute of Public Health, Tokyo, Japan (M. Mitobe, H. Kubota, K. Kobayashi, H. Miyake, J. Suzuki, K. Sadamasu); National Center for Global Health and Medicine, Tokyo (M. Takano, D. Mizushima, H. Gatanaga, S. Oka)

**Keywords:** *Chlamydia trachomatis*, men who have sex with men, multilocus sequence typing, outer membrane protein A, polymorphic membrane protein, phylogenetic analysis, Japan, bacteria, sexually transmitted infections

## Abstract

Several *Chlamydia trachomatis* lineages identified through outer membrane protein A genotyping or multilocus sequence typing have been circulating worldwide among men who have sex with men. In a study in Tokyo, Japan, we demonstrate that such lineages commonly belong to a specific polymorphic membrane protein E clade across genotypes.

*Chlamydia trachomatis* infection is the most common sexually transmitted infection (STI) worldwide. Because most infections are asymptomatic, sexual transmission generally occurs without notification. This aspect of transmission creates a risk for persistent undiagnosed *C. trachomatis* infection, which can lead to ascending infection in the female genital tract and result in serious conditions, such as pelvic inflammatory disease, ectopic pregnancy, and infertility.

The standard epidemiologic marker used for *C. trachomatis* genotyping is *ompA*, which encodes the major outer membrane protein. *C. trachomatis* is classified into 18 genotypes on the basis of *ompA* diversity, and the genotypes are further categorized into 3 groups on the basis of their predominant anatomic sites: ocular (A–C), urogenital and anorectal (D–K), and lymphogranuloma venereum (L1–L3). The molecular epidemiology of *C. trachomatis* is characterized by the predominance of *ompA* genotypes D, G, and J among men who have sex with men (MSM) in many countries (*1*–*8*). Multilocus sequence typing (MLST) has revealed that MSM-specific sequence types (STs) are present in these genotypes (*5*–*8*) and that those STs are distributed globally, suggesting the presence of specific international transmission networks among MSM. However, how the specific STs were selectively disseminated among MSM across several *ompA* genotypes or whether they have any shared underlying characteristics are unclear. The purpose of this study was to characterize the molecular epidemiology of *C. trachomatis* among MSM in Tokyo, Japan.

## The Study

We focused on *C. trachomatis* polymorphic membrane protein (Pmp) variation, which is considered to play a key role in the initial infection process (*9*,*10*). Among 9 Pmp groups (PmpA–PmpH) PmpE is the most diverse, and a specific clade has been identified in rectal samples from MSM (*11*), suggesting a potential target for molecular epidemiologic studies of *C. trachomatis*.

The clinical specimens were collected from MSM at an outpatient clinic at the National Center for Global Health and Medicine in Tokyo that specializes in providing care for MSM. We collected 7,200 pharyngeal and 1,904 urogenital specimens during October 2018–March 2021, and collected 703 rectal specimens during April 2019–March 2021. The men were participants in an HIV-negative cohort study on implementation of preexposure prophylaxis. The specimen collection methods have been described previously (*12*). In addition, 200 urogenital specimens and 42 cervical specimens were collected as non-MSM samples from outpatients attending general clinics (not specifically for MSM) with urinary or genital tract infections during the same period. The major departments of those clinics were obstetrics and gynecology (clinic A), gastroenterology (clinic B), and urology (clinic C). We selected patients who had clinically suspected *Neisseria gonorrhoeae* or *C. trachomatis* infection. This study was approved by the ethics committee of the Tokyo Metropolitan Institute of Public Health (approval no. 3KENKENKENDAI465GOU).

We sequenced the *C. trachomatis*–positive specimens, confirmed using an Aptima Combo 2 transcription-mediated amplification test (Hologic, https://www.hologic.com), to determine the *ompA* genotypes, as described previously (*13*). We performed MLST targeting 5 regions (*hctB*, *CT058*, *CT144*, *CT172*, and *pbpB*) using the Uppsala scheme as described in the PubMLST website (https://pubmlst.org/organisms/chlamydiales-spp), assigning new STs when they were discovered. On the basis of the determined STs, we constructed a minimum-spanning tree using the GrapeTree tree visualization program (*14*) with the MSTreeV2 algorithm. We amplified the near–full length of PmpE-encoding regions (2740 bp), which includes 5 variable regions (*11*), by nested PCR using primer sets. We used pmpE_1st_F (5′-GAAAAAAGCGTTTTTCTTTTTCCTTATCG-3′) and pmpE_1st_R (5′-TCCCCATTGAGATAATTACAGAAGGTTGA-3′) for the first PCR and used pmpE_2nd_F (5′-AACTCAGTTCCAGATCCTACGAAAGAGTC-3′) and pmpE_2nd_R (5′-ACTGGAAATGGAGAGTTAACCAACTCAAAG-3′) for the second PCR. We sequenced the PCR products through amplicon sequencing using MiSeq (Illumina, https://www.illumina.com). We constructed a nonrooted phylogenetic tree with the neighbor-joining method on the basis of the amino acid differences with MEGA7 software (https://www.megasoftware.net) using the amino acid sequences (907 aa) obtained by computational translation of DNA sequences corresponding to nucleotide numbers 1,025,723–1,028,443 of the *C. trachomatis* D/UW-3/CX genome (AE001273) (*15*).

**Figure 1:**
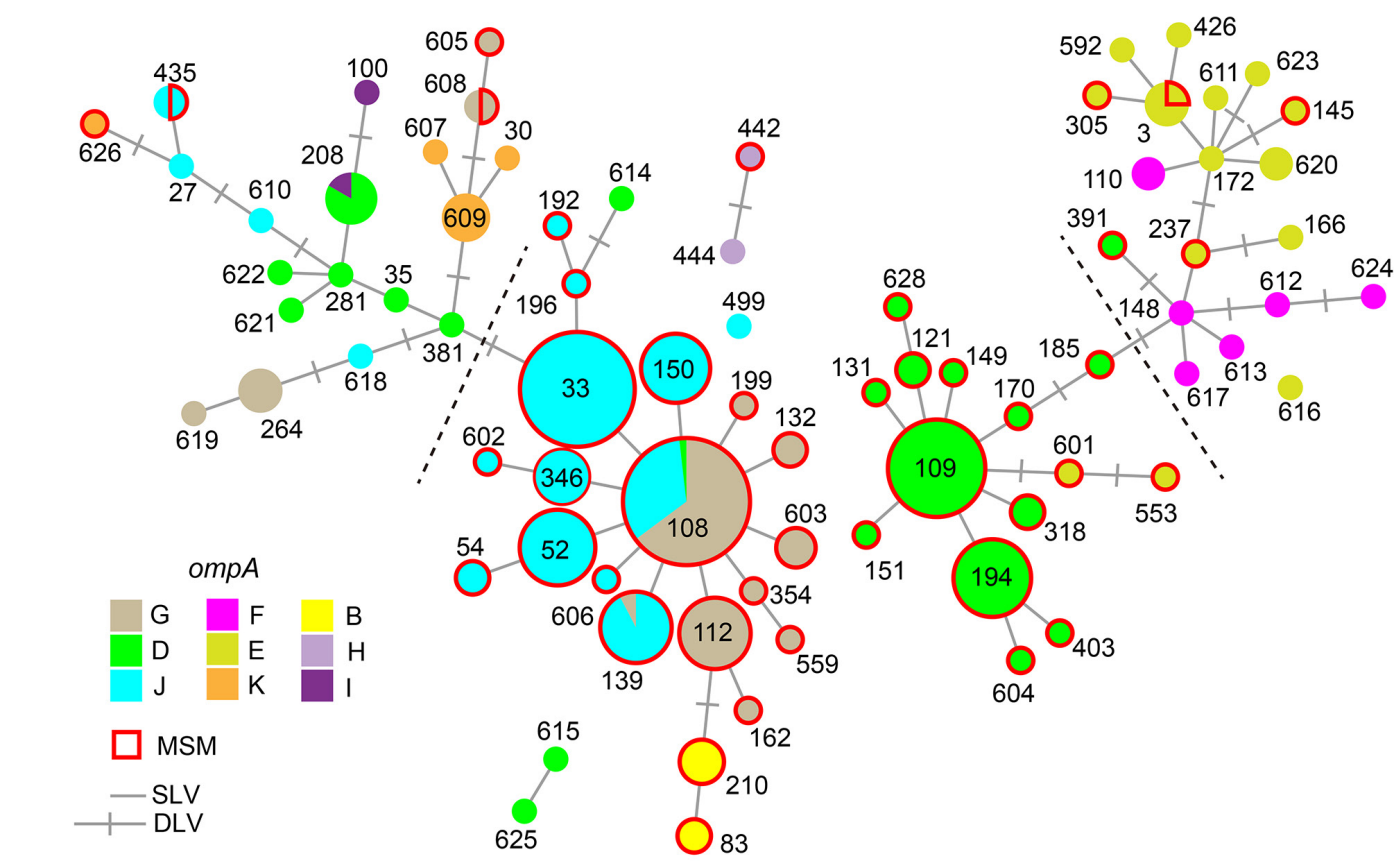
Minimum spanning tree based on sequence types (STs) and *ompA* of 298 *Chlamydia trachomatis* samples in study of clustering of specific polymorphic membrane protein E clade in *C. trachomatis* lineages from MSM, Japan. Each node indicates the ST number. SLVs and DLVs are linked. Samples from MSM are outlined in red, reflecting the proportion of samples in each node. The colors represent the *ompA* genotype. Nodes that contain several genotypes are shown as pie charts. Dashed lines are the assumed borders between the MSM and non-MSM lineages. DLV, double-locus variant; MSM, men who have sex with men; SLV, single-locus variant.

We fully analyzed a total of 298 *C. trachomatis*–positive specimens (245 from MSM and 53 from non-MSM) with *ompA* genotyping, MLST, and PmpE sequencing (Appendix 1). Although specimens were repeatedly collected from several MSM participants on different dates, no duplicate data (identical ST detection from the same site on different collection dates) were collected. The predominant *ompA* genotypes in the MSM population were D, G, and J (Figure 1; Appendix 2 Table 1), as previously reported in several countries (*5*–*8*). The most frequently detected STs were ST108 and its single-locus variants (SLVs) (e.g., ST33, ST52) and ST109 and its SLV (e.g., ST194). The main *ompA* genotypes were G/J in the ST108 lineage and D in the ST109 lineage (Appendix 2 Table 2). ST108 and ST109 are quadruple-locus variants of each other.

**Figure 2:**
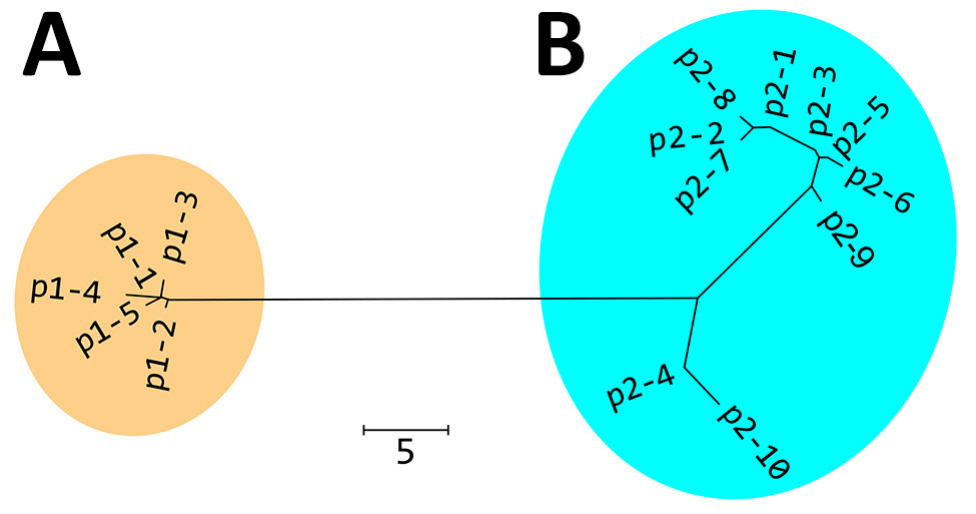
Nonrooted phylogenetic tree created on the basis of polymorphic membrane protein E of 298 *Chlamydia trachomatis* samples in study of clustering of specific polymorphic membrane protein E clade in *C. trachomatis* lineages from MSM, Japan. A) Cluster of 96.7% MSM (237 samples) and 0% non-MSM (0 samples); B) cluster of 3.3% MSM (8 samples) and 100% non-MSM (53 samples). p1 and p2 are 2 clades representing the MSM (p1) and non-MSM (p2) populations. The amino acid sequences of p1–1 to p2–10 are shown in Appendix 2 Figure. Numbers of samples included in each sequence: p1-1, n = 178; p1-2, n = 56; p1-3, n = 1; p1-4, n = 1; p1-5, n = 1; p2-1, n = 16; p2-2, n = 14; p2-3, n = 10; p2-4, n = 9; p2-5, n = 6; p2-6, n = 2; p2-7, n = 1; p2-8, n = 1; p2-9, n = 1; and p2-10, n = 1. Scale bar indicates the number of amino acid differences.

**Figure 3:**
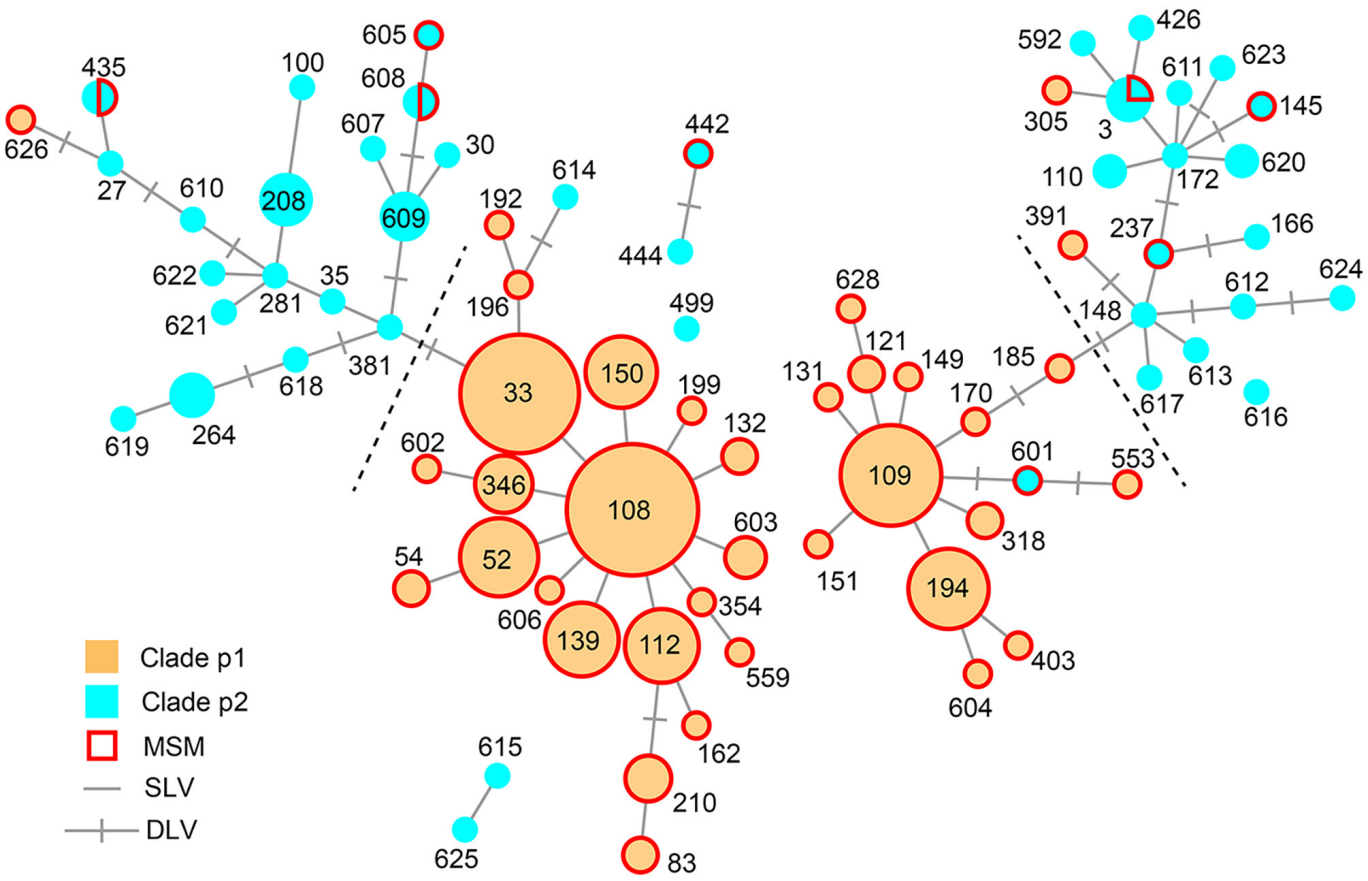
Minimum spanning tree based on sequence types (STs) and polymorphic membrane protein E (PmpE) of 298 *Chlamydia trachomatis* samples in study of clustering of specific PmpE clade in *C. trachomatis* lineages from MSM, Japan. Each node indicates the ST number. SLVs and DLVs are linked. Samples from MSM are outlined in red, reflecting the proportion of samples in each node. The PmpE clades p1 and p2 are colored using the same color codes as those used in Figure 2. Dashed lines are the assumed borders between the MSM and non-MSM lineages. DLV, double-locus variant; MSM, men who have sex with men; SLV, single-locus variant.

We detected 15 PmpE sequences in the 298 samples (p1–1 to p1–5 and p2–1 to p2–10), and those were clearly separated into 2 clades (named as p1 and p2) reflecting the MSM and non-MSM populations (Figure 2; Appendix 2 Figure). A few MSM samples were classified as p2, whereas no non-MSM samples were classified as p1. In MSM, the prevalence of the p1 clade did not differ significantly in urogenital, pharyngeal, and rectal samples (p = 0.141 by Fisher exact test) (Table), suggesting that the difference in clade between MSM and non-MSM samples was not attributable to differences in the anatomic sample collection sites. To investigate the phylogenetic relationships between the PmpE clades, we created a minimum spanning tree from STs showing the relationship to p1 and p2 (Figure 3). We further divided both lineages into likely sublineages corresponding to the difference in the PmpE clades (Figures 1, 3).

**Table:** Detected polymorphic membrane protein E clades of *Chlamydia trachomatis* according to study population and anatomic source of sample in study of *C. trachomatis* lineages from MSM, Japan*

Sample type	No. (%) samples		
	p1	p2	Total
MSM samples			
Rectal	201 (97.6)	5 (2.4)	206 (100)
Pharyngeal	25 (93)	2 (7)	27 (100)
Urogenital	11 (92)	1 (8)	12 (100)
Total	237 (96.7)	8 (3.3)	245 (100)
Non-MSM samples			
Urogenital	0	35 (100)	35 (100)
Cervix	0	18 (100)	18 (100)
Total	0	53 (100)	53 (100)

*The proportions of p1 and p2 in the MSM population did not differ significantly among the rectal, pharyngeal, and urogenital specimens (p = 0.141 by Fisher exact test). MSM, men who have sex with men.

Tokyo is a capital city with a population of >10 million and is connected to the 2 largest international airports in Japan; therefore, the similarity of the genotype distribution observed in this study to that observed in other countries is not surprising. The predominant *C. trachomatis* lineages among MSM in this study were centered around ST108 and ST109. Of the samples from MSM in this study, 89.4% (219/245) were major STs or their SLVs had been previously reported among MSM in other countries (*5*–*8*), demonstrating that the circulating lineages among MSM in Tokyo were typical of STs circulating internationally in MSM populations. In contrast, none of the STs of non-MSM samples were classified as major MSM STs, suggesting that the samples from non-MSM patients in this study were not linked to STs circulating in the global MSM population.

## Conclusions

This study revealed that most MSM-associated *C. trachomatis* STs belonged to the specific PmpE clade p1. This finding indicates that nonsimplex *C. trachomatis* lineages with shared microbiological characteristics involved in the infection process (*9*,*10*) likely disseminated in parallel through international MSM networks and that those shared characteristics might be involved in the infection process and transmission. Taken together, this study demonstrates the importance of PmpE as a target for molecular epidemiologic investigation to clarify the dynamics of *C. trachomatis* transmission.

Appendix 1 Additional data from study of clustering of polymorphic membrane protein E clade in *Chlamydia trachomatis* lineages from men who have sex with men, Japan.

Appendix 2 Additional information about clustering of polymorphic membrane protein E clade in *Chlamydia trachomatis* lineages from men who have sex with men, Japan.

## References

[1] Klint M, Löfdahl M, Ek C, Airell A, Berglund T, Herrmann B. Lymphogranuloma venereum prevalence in Sweden among men who have sex with men and characterization of *Chlamydia trachomatis ompA* genotypes. J Clin Microbiol. 2006;44:4066–71. 10.1128/JCM.00574-06 16971651 PMC1698335

[2] Twin J, Moore EE, Garland SM, Stevens MP, Fairley CK, Donovan B, et al. *Chlamydia trachomatis* genotypes among men who have sex with men in Australia. Sex Transm Dis. 2011;38:279–85. 10.1097/OLQ.0b013e3181fc6944 21085058

[3] Li JH, Cai YM, Yin YP, Hong FC, Shi MQ, Feng TJ, et al. Prevalence of anorectal *Chlamydia trachomatis* infection and its genotype distribution among men who have sex with men in Shenzhen, China. Jpn J Infect Dis. 2011;64:143–6. 10.7883/yoken.64.143 21519129

[4] Qin X, Zheng H, Xue Y, Ren X, Yang B, Huang J, et al. Prevalence of *Chlamydia trachomatis* genotypes in men who have sex with men and men who have sex with women using multilocus VNTR analysis—*ompA* typing in Guangzhou, China. PLoS One. 2016;11:e0159658. 10.1371/journal.pone.0159658 27434536 PMC4951006

[5] Piñeiro L, Villa L, Salmerón P, Maciá MD, Otero L, Vall-Mayans M, et al. Genetic characterization of non-lymphogranuloma venereum *Chlamydia trachomatis* indicates distinct infection transmission networks in Spain. Int J Mol Sci. 2023;24:6941. 10.3390/ijms24086941 37108105 PMC10138622

[6] Bom RJ, van der Helm JJ, Schim van der Loeff MF, van Rooijen MS, Heijman T, Matser A, et al. Distinct transmission networks of *Chlamydia trachomatis* in men who have sex with men and heterosexual adults in Amsterdam, The Netherlands. PLoS One. 2013;8:e53869. 10.1371/journal.pone.0053869 23342025 PMC3547048

[7] Herrmann B, Isaksson J, Ryberg M, Tångrot J, Saleh I, Versteeg B, et al. Global multilocus sequence type analysis of *Chlamydia trachomatis* strains from 16 countries. J Clin Microbiol. 2015;53:2172–9. 10.1128/JCM.00249-15 25926497 PMC4473235

[8] Versteeg B, Bruisten SM, van der Ende A, Pannekoek Y. Does typing of *Chlamydia trachomatis* using housekeeping multilocus sequence typing reveal different sexual networks among heterosexuals and men who have sex with men? BMC Infect Dis. 2016;16:162. 10.1186/s12879-016-1486-2 27090402 PMC4836166

[9] Debrine AM, Karplus PA, Rockey DD. A structural foundation for studying chlamydial polymorphic membrane proteins. Microbiol Spectr. 2023;11:e0324223. 10.1128/spectrum.03242-23 37882824 PMC10715098

[10] Favaroni A, Hegemann JH. *Chlamydia trachomatis* polymorphic membrane proteins (Pmps) form functional homomeric and heteromeric oligomers. Front Microbiol. 2021;12:709724. 10.3389/fmicb.2021.709724 34349750 PMC8326573

[11] Suchland RJ, Carrell SJ, Ramsey SA, Hybiske K, Debrine AM, Sanchez J, et al. Genomic analysis of MSM rectal *Chlamydia trachomatis* isolates identifies predicted tissue-tropic lineages generated by intraspecies lateral gene transfer-mediated evolution. Infect Immun. 2022;90:e0026522. 10.1128/iai.00265-22 36214558 PMC9670952

[12] Mizushima D, Takano M, Uemura H, Yanagawa Y, Aoki T, Watanabe K, et al. High prevalence and incidence of rectal *Chlamydia* infection among men who have sex with men in Japan. PLoS One. 2019;14:e0220072. 10.1371/journal.pone.0220072 31821348 PMC6903740

[13] Yoshida H, Kishi Y, Shiga S, Inoue S, Hagiwara T. Serotyping of *Chlamydia trachomatis* by polymerase chain reaction [in Japanese]. Sex Transm Infect. 1995;6:40–5.

[14] Zhou Z, Alikhan NF, Sergeant MJ, Luhmann N, Vaz C, Francisco AP, et al. GrapeTree: visualization of core genomic relationships among 100,000 bacterial pathogens. Genome Res. 2018;28:1395–404. 10.1101/gr.232397.117 30049790 PMC6120633

[15] Kumar S, Stecher G, Tamura K. MEGA7: Molecular Evolutionary Genetics Analysis version 7.0 for bigger datasets. Mol Biol Evol. 2016;33:1870–4. 10.1093/molbev/msw054 27004904 PMC8210823

