# Supplementary material for: Clustering of Polymorphic Membrane Protein E Clade in Chlamydia trachomatis Lineages from Men Who Have Sex with Men
Source: Emerg Infect Dis. 2024 Oct;30(10):2183–7. doi: 10.3201/eid3010.240852 (PMC11431897; doi:10.3201/eid3010.240852)
Supplement: Appendix 2 — Additional information about clustering of polymorphic membrane protein E clade in Chlamydia trachomatis lineages from men who have sex with men, Japan. [file 24-0852-Techapp-s2.pdf]

Article DOI: https://doi.org/10.3201/eid3010.240852

EID cannot ensure accessibility for supplementary materials supplied by authors. Readers who have difficulty accessing supplementary content should contact the authors for assistance.

Clustering of a Polymorphic Membrane Protein E Clade in *Chlamydia trachomatis* Lineages from Men who have Sex with Men

Appendix 2

Appendix 2 Table 1. Summary of the *ompA* genotypes detected according to the study population and anatomic site*

Category	Genotype									
	**G**	**D**	**J**	F	E	K	B	H	I	Total
MSM										
Rectal	**95 (46.1%)**	**49 (23.8%)**	**52 (25.2%)**	0 (0%)	5 (2.4%)	1 (0.5%)	4 (1.9%)	0 (0%)	0 (0%)	206 (100%)
Pharyngeal	**12 (44%)**	**6 (22%)**	**7 (26%)**	0 (0%)	0 (0%)	0 (0%)	1 (4%)	1 (4%)	0 (0%)	27 (100%)
Urogenital	**6 (50%)**	**3 (25%)**	**1 (8%)**	0 (0%)	1 (8%)	0 (0%)	1 (8%)	0 (0%)	0 (0%)	12 (100%)
Total	**113 (46.1%)**	**58 (23.7%)**	**60 (24.5%)**	0 (0.0%)	6 (2.4%)	1 (0.4%)	6 (2.4%)	1 (0.4%)	0 (0.0%)	245 (100%)
Non-MSM										
Urogenital	**3 (9%)**	**11 (31%)**	**3 (9%)**	6 (17%)	7 (20%)	4 (20%)	0 (0%)	1 (3%)	0 (0%)	35 (100%)
Cervix	**1 (6%)**	**3 (17%)**	**3 (17%)**	1 (6%)	5 (28%)	3 (17%)	0 (0%)	0 (0%)	2 (11%)	18 (100%)
Total	**4 (7.5%)**	**14 (26%)**	**6 (11%)**	7 (13%)	12 (23%)	7 (13%)	0 (0%)	1 (2%)	2 (4%)	53 (100%)

*The proportions of each genotype in the collection source are shown in parentheses. The numbers for genotypes G, D, and J are indicated in bold.

Appendix 2 Table 2. STs detected in this study and major STs detected in previous studies*

MSM/Non-MSM	ST	n	*ompA* (n)	PmpE	Comments and findings in previous studies†
MSM	ST108	55	J (35), G (19), D (1)	p1	Major MSM ST (*1–4*)
	ST33	42	G (42)	p1	Major MSM ST (*1*,*2*,*4*)
	ST109	28	D (28)	p1	Major MSM ST (*1–4*)
	ST194	16	D (16)	p1	SLV of MSM ST109 and ST571
	ST52	14	G (14)	p1	Major MSM ST (*1–4*)
	ST112	13	J (13)	p1	SLV of MSM ST108
	ST139	13	G (12), J (1)	p1	SLV of MSM ST108
	ST150	12	G (12)	p1	SLV of MSM ST33 and ST108
	ST346	7	G (7)	p1	SLV of MSM ST33 and ST108
	ST210	4	B (4)	p1	
	ST603	3	J (3)	p1	SLV of MSM ST108
	ST54	2	G (2)	p1	SLV of MSM ST52
	ST83	2	B (2)	p1	
	ST121	2	D (2)	p1	SLV of MSM ST109
	ST132	2	J (2)	p1	SLV of MSM ST108
	ST318	2	D (2)	p1	SLV of MSM ST109
	ST3	1	E (1)	p2	
	ST131	1	D (1)	p1	SLV of MSM ST109
	ST145	1	E (1)	p2	
	ST149	1	D (1)	p1	SLV of MSM ST109
	ST151	1	D (1)	p1	SLV of MSM ST109
	ST162	1	J (1)	p1	
	ST170	1	D (1)	p1	SLV of MSM ST109
	ST185	1	D (1)	p1	
	ST192	1	G (1)	p1	
	ST196	1	G (1)	p1	SLV of MSM ST33
	ST199	1	J (1)	p1	SLV of MSM ST108
	ST237	1	E (1)	p2	
	ST305	1	E (1)	p1	
	ST354	1	J (1)	p1	SLV of MSM ST33 and ST108
	ST391	1	D (1)	p1	
	ST403	1	D (1)	p1	

MSM/Non-MSM	ST	n	*ompA* (n)	PmpE	Comments and findings in previous studies†
	ST435	1	G (1)	p2	
	ST442	1	H (1)	p2	
	ST553	1	E (1)	p1	
	ST559	1	J (1)	p1	
	ST601	1	E (1)	p1	
	ST602	1	G (1)	p1	
	ST604	1	D (1)	p1	
	ST605	1	J (1)	p2	
	ST606	1	G (1)	p1	SLV of ST33, ST58, and ST108
	ST608	1	J (1)	p2	
	ST626	1	K (1)	p1	
	ST628	1	D (1)	p1	
Non-MSM	ST208	6	D (5), I (1)	p2	
	ST609	5	K (5)	p2	
	ST264	4	J (4)	p2	
	ST3	3	E (3)	p2	
	ST110	2	F (2)	p2	
	ST620	2	E (2)	p2	
	ST27	1	G (1)	p2	
	ST30	1	K (1)	p2	
	ST35	1	D (1)	p2	
	ST100	1	I (1)	p2	
	ST148	1	F (1)	p2	
	ST166	1	E (1)	p2	
	ST172	1	E (1)	p2	
	ST281	1	D (1)	p2	
	ST381	1	D (1)	p2	
	ST426	1	E (1)	p2	
	ST435	1	G (1)	p2	
	ST444	1	H (1)	p2	
	ST499	1	D (1)	p2	
	ST592	1	E (1)	p2	
	ST607	1	K (1)	p2	
	ST608	1	J (1)	p2	

MSM/Non-MSM	ST	n	*ompA* (n)	PmpE	Comments and findings in previous studies†
	ST610	1	G (1)	p2	
	ST611	1	E (1)	p2	
	ST612	1	F (1)	p2	
	ST613	1	F (1)	p2	
	ST614	1	D (1)	p2	
	ST615	1	D (1)	p2	
	ST616	1	E (1)	p2	
	ST617	1	F (1)	p2	
	ST618	1	G (1)	p2	
	ST619	1	J (1)	p2	
	ST621	1	D (1)	p2	
	ST622	1	D (1)	p2	
	ST623	1	E (1)	p2	
	ST624	1	F (1)	p2	
	ST625	1	D (1)	p2	
References‡					
MSM	ST33				Major MSM ST (*1*,*2*,*4*)
	ST52				Major MSM ST (*1–4*)
	ST58				Major MSM ST (*2,3,4*)
	ST108				Major MSM ST (*1–4*)
	ST109				Major MSM ST (*1–4*)
	ST571				Major MSM ST (*1*)

*MSM, men who have sex with men; PmpE, polymorphic membrane protein E; SLV, single-locus variant; ST, sequence type.

†Major STs in References 1–4 and SLVs are shown.

‡References 1 and 3 declared major STs in their text, whereas the top 5 STs in References 2 and 4 were picked up as their major STs for MSM.

Appendix 2 Figure (following pages). Sequence alignments of PmpE. The numbers at the right ends indicate the amino acid number of *Chlamydia trachomatis* D/UW-3/CX PmpE (AE001273). Variable regions (VRs) 1 to 5 as described previously (*5*) are shown.

```
D/UW-3/CX   DPTKESLSNKISLTGDTHNLTNCYLDNLRYILAILQKTPNEGAAVTITDYLSFFDTQKEGIYFAKNLTPESGGAIGYASP 113
p1-1        ....................................R...........................................
p1-2        ....................................R...........................................
p1-3        ........................V...........R...........................................
p1-4        ....................................R...........................................
p1-5        ....................................R...........................................
p2-1        ................................................................................
p2-2        ................................................................................
p2-3        ................................................................................
p2-4        ....................................R...........................................
p2-5        ................................................................................
p2-6        ................................................................................
p2-7        ................................................................................
p2-8        ................................................................................
p2-9        ................................................................................
p2-10       ....................................R...........................................
```

VR1

```
D/UW-3/CX   NSPTVEIRDTIGPVIFENNTCCRLFTWRNP-YAADKIREGGAIHAQNLYINHNHDVVGFMKNFSYVQGGAISTANTFVVS 192
p1-1        .......................P...S..RA..N.............................................
p1-2        .......................P...S..RA..N.............................................
p1-3        .......................P...S..RA..N.............................................
p1-4        .......................P...S..RA..N.............................................
p1-5        .......................P...S..RA..N.............................................
p2-1        ..............................-.................................................
p2-2        ..............................-.................................................
p2-3        ..............................-.................................................
p2-4        .......................P......LT.VN.............................................
p2-5        ..............................-.................................................
p2-6        ..............................-.................................................
p2-7        ..............................-...................................R.............
p2-8        ..............................-.................................................
p2-9        ...........................S..RA..N.............................................
p2-10       .......................P......LT.VN.............................................
```

D/UW-3/CX	ENQSCFLFMDNICIQTNTAGKGGAIYAGTSNSFESNNCDLFFINNACCAGGAIFSPICSLTGNRGNIVFYNNRCFKNVET	272
p1-1	....	
p1-2	....	
p1-3	....	
p1-4	....I...................	
p1-5	....	
p2-1	....	
p2-2	....	
p2-3	....	
p2-4	....	
p2-5	....	
p2-6	....	
p2-7	....	
p2-8	....	
p2-9	....	
p2-10	....I..	

D/UW-3/CX	ASSEASDGGAIKVTTRLDVTGNRGRIFFSDNITKNYGGAIYAPVVTLVDNGPTYFINNIANNKGGAIYIDGTSNSKISAD	352
p1-1	....E.................	
p1-2	....E.................	
p1-3	....E.................	
p1-4	....E.................	
p1-5	....E.................	
p2-1	....	
p2-2	....	
p2-3	....	
p2-4	....E.................	
p2-5	....	
p2-6	....	
p2-7	....	
p2-8	..................................D...	
p2-9	....	
p2-10	.....P..E.................	

VR2

```
D/UW-3/CX   RHAIIFNENIVTNVTNANGTSTSANPPRRNAITVASSSGEILLGAGSSQNLIFYDPIEVSNAGVSVSFNKEADQTGSVVF 432
p1-1        ..............SND.I..--...........T......I....EG...........-......F.............
p1-2        ..............SND.I..--...........T......I....EG...........-......F.............
p1-3        ..............SND.I..--...........T......I....EG...........-......F.............
p1-4        ..............SND.I..--...........T......I....EG...........-......F.............
p1-5        ..T...........SND.I..--...........T......I....EG...........-......F.............
p2-1        ................................................................................
p2-2        ................................................................................
p2-3        ................................................................................
p2-4        ...........................................................-......F.............
p2-5        ................................................................................
p2-6        ................................................................................
p2-7        ................................................................................
p2-8        ................................................................................
p2-9        ................................................................................
p2-10       ...........................................................-......F.............
```

VR3

```
D/UW-3/CX   SGATVNSADFHQRNLQTKTPAPLTLSNGFLCIEDHAQLTVNRFTQTGGVVSLGNGAVLSCYKNGTGDSASNASITLKHIG 512
p1-1        .....................T...NH...................K...A............----NST...V......
p1-2        .....................T...NH...................K...A............----NST...V......
p1-3        .....................T...NH...................K...A............----NST...V......
p1-4        .....................T...NH...................K...A............----NST...V......
p1-5        .....................T...NH...................K...A............----NST...V......
p2-1        ................................................................................
p2-2        ................................................................................
p2-3        ................................................................................
p2-4        ................................................................................
p2-5        ................................................................................
p2-6        ................................................................................
p2-7        ................................................................................
p2-8        ................................................................................
p2-9        ................................................................................
p2-10       ................................................................................
```

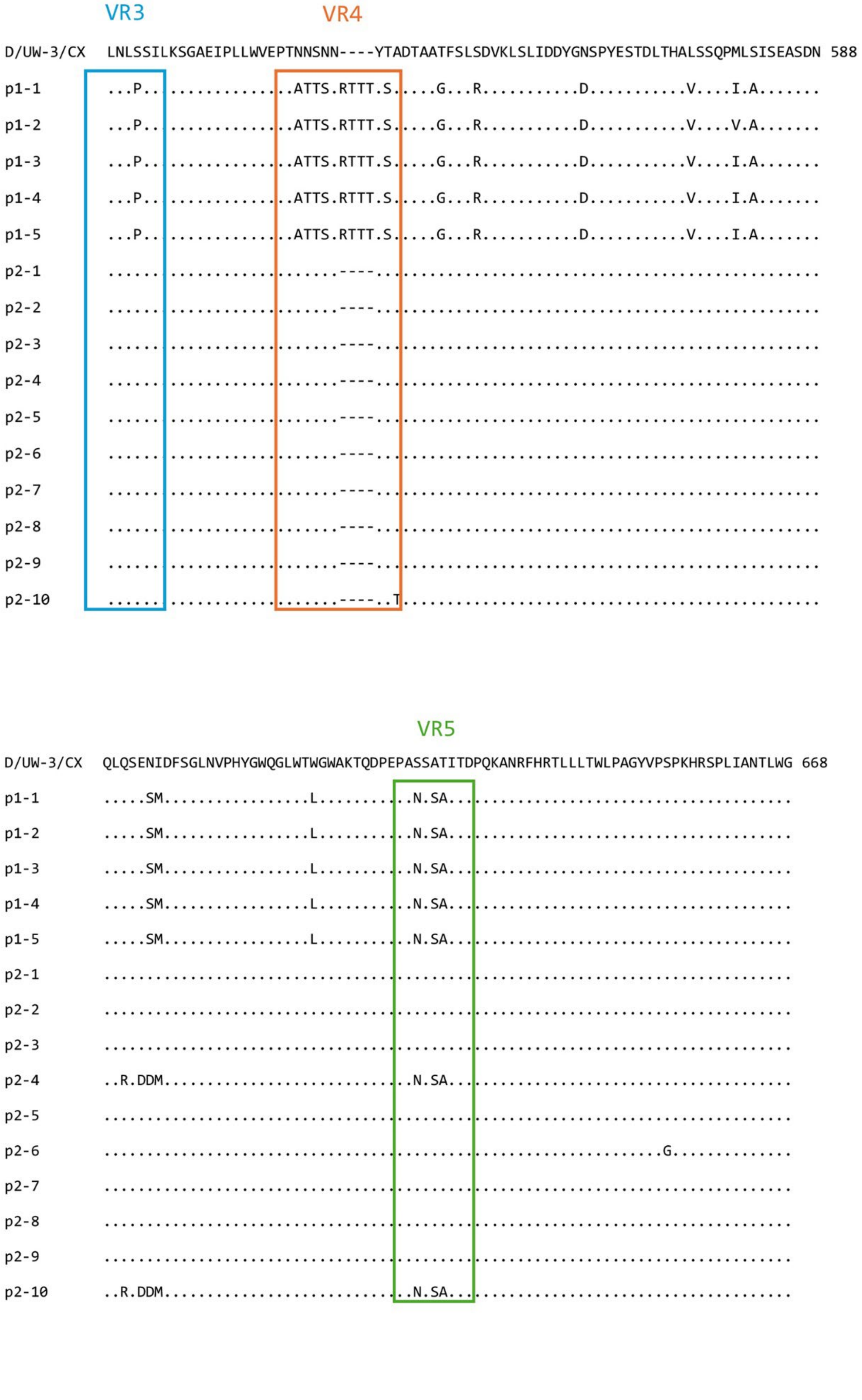
VR3
VR4
D/UW-3/CX LNLSSILKSGAEIPLLWVEPTNNSNN----YTADTAATFSLSDVKLSLIDDYGNSPYESTDLTHALSSQPMLSISEASDN 588
p1-1 ...P.................ATTS.RTTT.S.....G...R...........D...........V....I.A.......
p1-2 ...P.................ATTS.RTTT.S.....G...R...........D...........V....V.A.......
p1-3 ...P.................ATTS.RTTT.S.....G...R...........D...........V....I.A.......
p1-4 ...P.................ATTS.RTTT.S.....G...R...........D...........V....I.A.......
p1-5 ...P.................ATTS.RTTT.S.....G...R...........D...........V....I.A.......
p2-1 ....----..
p2-2 ....----..
p2-3 ....----..
p2-4 ....----..
p2-5 ....----..
p2-6 ....----..
p2-7 ....----..
p2-8 ....----..
p2-9 ....----..
p2-10 ..........................----..T...
VR5
D/UW-3/CX QLQSENIDFSGLNVPHYGWQGLWTWGWAKTQDPEPASSATITDPQKANRFHRTLLLTWLPAGYVPSPKHRSPLIANTLWG 668
p1-1 .....SM.................L...........N.SA..
p1-2 .....SM.................L...........N.SA..
p1-3 .....SM.................L...........N.SA..
p1-4 .....SM.................L...........N.SA..
p1-5 .....SM.................L...........N.SA..
p2-1 ....
p2-2 ....
p2-3 ....
p2-4 ..R.DDM.............................N.SA..
p2-5 ....
p2-6 ....G..............
p2-7 ....
p2-8 ....
p2-9 ....
p2-10 ..R.DDM.............................N.SA..

```
D/UW-3/CX   NMLLATESLKNSAELTPSGHPFWGITGGGLGMMVYQDPRENHPGFHMRSSGYSAGMIAGQTHTFSLKFSQTYTKLNERYA 748
p1-1        ..................D.............................................................
p1-2        ..................D.............................................................
p1-3        ..................D.............................................................
p1-4        ..................D.............................................................
p1-5        ..................D.............................................................
p2-1        ................................................................................
p2-2        ................................................................................
p2-3        ................................................................................
p2-4        ..................D.............................................................
p2-5        ..................D.............................................................
p2-6        ..................D.............................................................
p2-7        ................................................................................
p2-8        ................................................................................
p2-9        ................................................................................
p2-10       ..................D.............................................................
```

```
D/UW-3/CX   KNNVSSKNYSCQGEMLFSLQEGFLLTKLVGLYSYGDHNCHHFYTQGENLTSQGTFRSQTMGGAVFFDLPMKPFGSTHILT 828
p1-1        .........................A......................................................
p1-2        .........................A......................................................
p1-3        .........................A......................................................
p1-4        .........................A......................................................
p1-5        .........................A......................................................
p2-1        ................................................................................
p2-2        ................................................................................
p2-3        .........................A......................................................
p2-4        .........................A......................................................
p2-5        .........................A......................................................
p2-6        .........................A......................................................
p2-7        ................................................................................
p2-8        ................................................................................
p2-9        .........................A......................................................
p2-10       .........................A......................................................
```

```
D/UW-3/CX  APFLGALGIYSSLSHFTEVGAYPRSFSTKTPLINVLVPIGVKGSFMNATHRPQAWTVELAYQPVLYRQEPGIAAQLLASK 908
p1-1       .................................................Q...................L...T......
p1-2       .................................................Q...................L...T......
p1-3       .................................................Q...................L...T......
p1-4       .................................................Q...................L...T.....Q
p1-5       .................................................Q...................L...T......
p2-1       .........................................................................T......
p2-2       ................................................................................
p2-3       .................................................Q...................L...T......
p2-4       .................................................Q...................L...T......
p2-5       .................................................Q...................L...T......
p2-6       .................................................Q...................L...T......
p2-7       ................................................................................
p2-8       ................................................................................
p2-9       .................................................Q...................L...T......
p2-10      .................................................Q...................L...T......
```

```
D/UW-3/CX   GIWFGSGSPSSRHAMSYKISQQTQPLSWLTLH 940
p1-1        ................................
p1-2        ................................
p1-3        ................................
p1-4        ................................
p1-5        ................................
p2-1        ................................
p2-2        ................................
p2-3        ................................
p2-4        ................................
p2-5        ................................
p2-6        ................................
p2-7        ................................
p2-8        ................................
p2-9        ................................
p2-10       ................................
```

References

<jrn>1. Piñeiro L, Villa L, Salmerón P, Maciá MD, Otero L, Vall-Mayans M, et al. Genetic characterization of non-lymphogranuloma venereum *Chlamydia trachomatis* indicates distinct infection transmission networks in Spain. Int J Mol Sci. 2023;24:6941. PubMed https://doi.org/10.3390/ijms24086941</jrn>

<jrn>2. Bom RJ, van der Helm JJ, Schim van der Loeff MF, van Rooijen MS, Heijman T, Matser A, et al. Distinct transmission networks of *Chlamydia trachomatis* in men who have sex with men and heterosexual adults in Amsterdam, The Netherlands. PLoS One. 2013;8:e53869. PubMed https://doi.org/10.1371/journal.pone.0053869</jrn>

<jrn>3. Herrmann B, Isaksson J, Ryberg M, Tångrot J, Saleh I, Versteeg B, et al. Global multilocus sequence type analysis of *Chlamydia trachomatis* strains from 16 countries. J Clin Microbiol. 2015;53:2172–9. PubMed https://doi.org/10.1128/JCM.00249-15</jrn>

<jrn>4. Versteeg B, Bruisten SM, van der Ende A, Pannekoek Y. Does typing of *Chlamydia trachomatis* using housekeeping multilocus sequence typing reveal different sexual networks among heterosexuals and men who have sex with men? BMC Infect Dis. 2016;16:162. PubMed https://doi.org/10.1186/s12879-016-1486-2</jrn>

<jrn>5. Suchland RJ, Carrell SJ, Ramsey SA, Hybiske K, Debrine AM, Sanchez J, et al. Genomic analysis of MSM rectal *Chlamydia trachomatis* isolates identifies predicted tissue-tropic lineages generated by intraspecies lateral gene transfer-mediated evolution. Infect Immun. 2022;90:e0026522. PubMed https://doi.org/10.1128/iai.00265-22</jrn>
